# Primary versus rescue laparoscopic ureterocalicostomy for complex upper urinary tract obstruction: a single-center series with long-term follow-up

**DOI:** 10.1007/s11255-026-05276-0

**Published:** 2026-07-19

**Authors:** Henri Luiz Morgan, Filipe de Arruda Pessoa, Eduardo Riccetto, Patrícia Rodrigues Candido, Victor Srougi, Francisco Tibor Dénes, William Carlos Nahas, Marco Antonio Arap

**Affiliations:** 1 Faculdade de Medicina da Universidade de São Paulo, Rua Capote Valente, 900, São Paulo, SP 05409-002 Brazil; 2 Hospital Sírio Libanês, São Paulo, Brazil

**Keywords:** Ureterocalicostomy, Upper ureteral stricture, Ureteropelvic junction obstruction, Ureteral reconstruction, Laparoscopy

## Abstract

**Purpose:**

To compare perioperative and long-term outcomes of laparoscopic ureterocalicostomy (UC) performed as a primary-versus-rescue reconstruction after failed prior repair in complex upper urinary tract obstruction.

**Methods:**

We retrospectively reviewed a prospectively maintained single-institution database of 24 patients who underwent laparoscopic UC between January 2008 and August 2024; UC was decided intraoperatively when conditions precluded safe pyeloplasty. The comparison was restricted to primary ureteropelvic junction obstruction (*n* = 23): rescue UC after failed prior repair (*n* = 12) versus primary UC without previous repair (*n* = 11). Demographics, operative data, complications, split renal function (DTPA scintigraphy), serum creatinine/eGFR, and symptom resolution were compared between and within groups.

**Results:**

The cohort was predominantly adult (33% younger than 18). The two groups were comparable in age, symptom duration, operative time, blood loss, and hospital stay (all *p* > 0.4). Pre- and postoperative split renal functions were comparable between groups (*p* = 0.35 and *p* = 0.97) and remained stable within both groups (40.0 ± 20.6% to 43.2 ± 18.5% overall). There were no conversions and only minor complications. At a median follow-up of 60 months, 22 of 24 patients (91.6%) achieved resolution of the obstruction. The two adverse outcomes were one primary failure (excluded patient, nephrectomy) and one late recurrence at 18 months. At last follow-up, 22 of 24 patients (91.6%) had durable resolution of the obstruction. There were two adverse outcomes of different nature: one primary failure (the excluded patient, who never improved and required nephrectomy) and one late recurrence in the primary group, in a patient with initial resolution who restenosed at 18 months; both related to complex calyceal anatomy.

**Conclusion:**

Laparoscopic UC provided durable long-term resolution of obstruction whether performed as a primary or a rescue reconstruction. UC is a reliable option in both unoperated and redo settings when pyeloplasty is unsafe, provided that calyceal anatomy is carefully assessed.

## Introduction

Ureterocalicostomy was first described by Neuwirt in 1948 for the treatment of ureteropelvic junction (UPJ) obstruction. The procedure involves resecting the lower-pole parenchyma, which is usually thinned by hydronephrosis, and anastomosing the spatulated ureter directly to the inferior renal calyx to restore efficient urinary drainage [1]. The first laparoscopic ureterocalicostomy was reported 2 decades ago, when Gill et al. described a two-case series [2], and the first robot-assisted procedure was reported by Korets et al. in 2007 [3].

To date, ureterocalicostomy remains an uncommon procedure. It has traditionally been regarded as a rescue or bail-out option after failed pyeloplasty [4–6], in which dissection and re-anastomosis are difficult because of extensive fibrotic scarring. Recognized indications also include UPJ obstruction with an intrarenal pelvis, high ureteral insertion, severe hydronephrosis, and horseshoe kidney, all with favorable reported outcomes [7, 8].

In 2014, we published our institution’s experience with laparoscopic ureterocalicostomy for complex upper urinary tract obstruction, which at the time was the largest series reported [9]. Since then, several centers have reported their laparoscopic and robotic results, confirming the safety and efficacy of the technique [10–13], and a recent review found success rates exceeding 90% in most series [6].

Nevertheless, the available evidence still consists mostly of case reports and small, single-institution retrospective series, and almost all describe only short- to mid-term follow-up [6, 9–13]. Two questions, therefore, remain insufficiently addressed: how durable the functional result is in the long term, and whether ureterocalicostomy performs as well when it is the first reconstruction of an unoperated obstruction (primary) as it does in its classic role after a failed repair (rescue). Here, we directly compare primary and rescue ureterocalicostomy in a single-institution series with long-term follow-up (median 60 months), to clarify whether the technique performs as well outside its classic rescue indication.

## Patients and methods

### Study design and definitions

We retrospectively reviewed a prospectively maintained database of all patients who underwent laparoscopic ureterocalicostomy for complex upper urinary tract obstruction at the authors’ center between January 2008 and August 2024. Preoperatively, obstruction was confirmed with renal scintigraphy and either contrast-enhanced computed tomography (CT; 22 patients) or intravenous urography (2 patients). Although ultrasonography and dynamic renal scintigraphy establish the diagnosis of obstruction, cross-sectional contrast imaging was obtained primarily to characterize the calyceal and pelvic anatomy and to plan the reconstruction; the single failure in this series, in a patient whose contrast allergy precluded a complete calyceal assessment, illustrates the importance of this step. All patients showed thin renal parenchyma, particularly at the lower pole, and were counseled on ureterocalicostomy as an alternative.

We defined complex upper urinary tract obstruction as ureteropelvic junction or proximal ureteral obstruction not amenable to a standard dismembered pyeloplasty—owing to a failed previous repair with dense fibrosis, a long proximal ureteral stricture, or unfavorable anatomy such as a high ureteral insertion with a malrotated kidney or a small intrarenal pelvis—together with thinning of the lower-pole parenchyma. There was no fixed numerical threshold for offering reconstruction rather than nephrectomy; the decision was individualized, based on the surgeon’s assessment of lower-pole parenchymal thinning on imaging and of the technical feasibility of a tension-free repair.

In every case, the decision to perform ureterocalicostomy was made intraoperatively. After mobilization and dissection, ureterocalicostomy was chosen when local conditions—intense fibrosis, a long ureteral stricture, or a small intrarenal pelvis with an unfavorable high insertion—precluded a safe, tension-free pyeloplasty or re-pyeloplasty.

Patient demographics, clinical presentation, symptom duration, laterality, operative time, estimated blood loss, hospital stay, complications, and clinical and functional outcomes were recorded. Postoperatively, obstruction was assessed clinically and by renal scintigraphy 6 weeks after removal of the double-J stent. Per institutional protocol, patients with no obstruction on the first postoperative scintigraphy did not undergo further contrast imaging, in order to limit radiation exposure; patients with persistent symptoms or inconclusive scintigraphy were re-evaluated two months later with CT or intravenous urography.

For analysis, patients with ureteropelvic junction obstruction were classified into two groups according to whether the obstruction had previously been surgically repaired. The rescue group (*n* = 12) comprised patients in whom ureterocalicostomy followed a failed previous reconstruction of the ureteropelvic junction (failed pyeloplasty or failed endopyelotomy). The primary group (*n* = 11) comprised patients with primary, previously unoperated ureteropelvic junction obstruction, in whom an unfavorable congenital anatomy (typically a high ureteral insertion with a malrotated kidney) led to ureterocalicostomy at the first operation. One further patient, with a secondary ureteropelvic junction stenosis after multiple prior urological procedures, is described as part of the overall cohort but was excluded from the primary-versus-rescue comparison, which was therefore restricted to primary ureteropelvic junction obstruction (*n* = 23). Restricting the comparison in this way also reduces the clinical heterogeneity of the compared groups.

Long-term surveillance followed a standardized institutional protocol: annual DTPA diuretic renography, at least one contrast-enhanced CT within the first year, and annual ultrasonography thereafter. Estimated GFR could not be calculated uniformly across the cohort: the pediatric subset lacked the height data required for the Schwartz equation, adult estimating equations are not validated in children, and longitudinal serum creatinine is confounded by growth-related increases in muscle mass.

Treatment success was defined a priori as the combination of (i) resolution or significant improvement of symptoms and (ii) resolution of the obstructive pattern on diuretic renography, i.e., conversion of an obstructive drainage curve to a non-obstructive or equivocal pattern; both criteria were required. Given the retrospective, multi-era nature of the renographic data, no fixed numerical T½ cut-off was applied. Differential renal function was analyzed separately and was not part of the success definition: function of the operated kidney was classified as improved or worsened when split function changed by at least 5 percentage points in the corresponding direction, and as stable otherwise.

### Surgical technique

Informed consent was obtained from all patients, who were counseled on the surgical risks, including bleeding, conversion, urinary diversion, and possible nephrectomy. Surgery was preceded by an 8-h fast and prophylactic intravenous cefalotin at induction.

The operative technique has been described in detail elsewhere, including a video published by our group [14], and is summarized briefly here. After general anesthesia and placement in a 45° lateral flank position, a transperitoneal three-port approach was used (a 10-mm umbilical camera port and two 5-mm working ports, with a fourth port added when needed); pneumoperitoneum was established with an open Hasson technique in patients with prior abdominal surgery and with a Veress needle otherwise. The colon was mobilized and the upper ureter dissected to the renal pelvis or the fibrotic segment; vascular clamping was not required, as the thin lower-pole parenchyma allowed hemostasis with cautery. A classic dismembered ureterocalicostomy was performed in 17 patients (ureteral transection and spatulation, lower-pole resection, antegrade double-J stenting, and a running 4–0 polyglycolic acid mucosa-to-mucosa anastomosis) and a termino-lateral technique without ureteral transection in 7. A closed suction drain was left lateral to the anastomosis; the drain and Foley catheter were removed at 48 h and the double-J stent at 6 weeks.

### Statistical analysis

Statistical analyses were performed with GraphPad Prism (version 11.0). The Shapiro–Wilk test was used to assess the normality of quantitative variables. Between-group comparisons were performed with the unpaired Student's t test for normally distributed variables and the Mann–Whitney test otherwise. A significance level of 5% (*p* ≤ 0.05) was adopted.

## Results

Twenty-four patients underwent laparoscopic ureterocalicostomy during the study period. Over the same interval, 638 pyeloplasties were performed at our institution, so ureterocalicostomy accounted for approximately 3.6% of upper-tract reconstructions for ureteropelvic junction obstruction. Twelve patients had a previous failed repair of the ureteropelvic junction—open pyeloplasty, repeat laparoscopic pyeloplasty, or endoscopic endopyelotomy—and were classified as the rescue group, whereas 11 had primary, previously unoperated obstruction (most often a high ureteral insertion with a malrotated kidney, and in one case an intrarenal pelvis) and formed the primary group; these 23 patients constituted the comparative analysis. The remaining patient had a secondary ureteropelvic junction stenosis after multiple prior stone procedures and was included in the overall cohort description but not in the comparison.

The cohort was predominantly adult: 16 patients (66.7%) were adults (≥ 18 years), 3 were adolescents (13–17), and 5 were children (< 13); 8 patients (33%) were younger than 18. The mean age was 28.3 years (median 27; range 2–64) and 19 patients (79.1%) were female. Almost all patients were symptomatic: 23 (95.8%) presented with lumbar pain and 7 (29.2%) with urinary tract infection; the only patient without pain (a 2-year-old) presented with urinary tract infection, and two patients had additional findings (one renal dysfunction, one nephrolithiasis). The near-absence of incidentally detected, asymptomatic obstruction reflects the tertiary referral nature of the cohort and its enrichment for complex and previously operated cases. The right side was treated in 15 patients (62.5%) (Table 1).

**Table 1:** Clinical and outcome data

Age	Group	Indication	Symptoms	Technique	Symptom improvement	Recurrence	Preop DTPA (%)	Postop DTPA (%)
62	Excl.*	Secondary UPJ stenosis	Pain	T-T	No	–	26	–
64	Rescue	Failed endopyelotomy	Pain	T-T	Yes	No	34	31
41	Rescue	Failed endopyelotomy	Pain	T-T	Yes	No	36	41
2	Rescue	Failed pyeloplasty	UTI	T-L	Yes	No	42	51
27	Rescue	Failed pyeloplasty	Pain + UTI	T-T	Yes	No	34	45
25	Rescue	Failed pyeloplasty	Pain + UTI	T-T	Yes	No	26	30
9	Rescue	Failed pyeloplasty	Pain + UTI	T-L	Yes	No	59	56
35	Rescue	Failed pyeloplasty	Pain	T-T	Yes	No	88	79
16	Rescue	Failed pyeloplasty	Pain	T-L	Yes	No	42	46
28	Rescue	Failed pyeloplasty	Pain + UTI	T-T	Yes	No	37	36
7	Rescue	Failed pyeloplasty	Pain	T-L	Yes	No	34	25
27	Rescue	Failed pyeloplasty	Pain + UTI	T-T	Yes	No	29	34
30	Rescue	Failed pyeloplasty	Pain	T-T	Yes	No	52	46
17	Primary	High UPJ + malrotated kidney	Pain	T-T	Yes	No	23	56
57	Primary	High UPJ + malrotated kidney	Pain	T-T	Yes	No	26	34
32	Primary	High UPJ + malrotated kidney	Pain + UTI	T-T	Yes	No	55	50
60	Primary	High UPJ + malrotated kidney	Pain	T-T	Yes	No	57	52
17	Primary	High UPJ + malrotated kidney	Pain	T-L	Yes	No	100	100
8	Primary	High UPJ + malrotated kidney	Pain	T-L	Yes	No	41	42
18	Primary	High UPJ + malrotated kidney	Pain	T-T	Yes	No	47	50
19	Primary	High UPJ + malrotated kidney	Pain	T-T	Yes	No	18	28
27	Primary	High UPJ + malrotated kidney	Pain	T-T	Yes	Yes	12	16
7	Primary	High UPJ + malrotated kidney	Pain	T-L	Yes	No	13	16
45	Primary	Intrarenal pelvis	Pain	T-T	Yes	No	30	29

Primary: ureterocalicostomy without previous repair of the obstruction; rescue: ureterocalicostomy after a failed previous repair

*DTPA* diethylenetriamine pentaacetic acid renal scintigraphy, *UPJ* ureteropelvic junction, *UTI* urinary tract infection, *T-T* termino-terminal (dismembered), *T-L* termino-lateral

*Excl.: a secondary ureteropelvic junction stenosis after multiple prior urological procedures that failed and required nephrectomy; included in the cohort description but excluded from the primary-versus-rescue comparison

Across the whole cohort, the mean operative time was 200 min (range 50–270), the mean estimated blood loss 84 mL (range 20–250), and the mean hospital stay 3.3 days (range 2–6). There were no conversions to open surgery and no intraoperative complications. Two postoperative complications occurred (Clavien I adynamic ileus in a pediatric patient, managed conservatively; Clavien III urinary tract infection treated with antibiotics and nephrostomy); no Clavien IV–V events occurred.

**Fig. 1:**
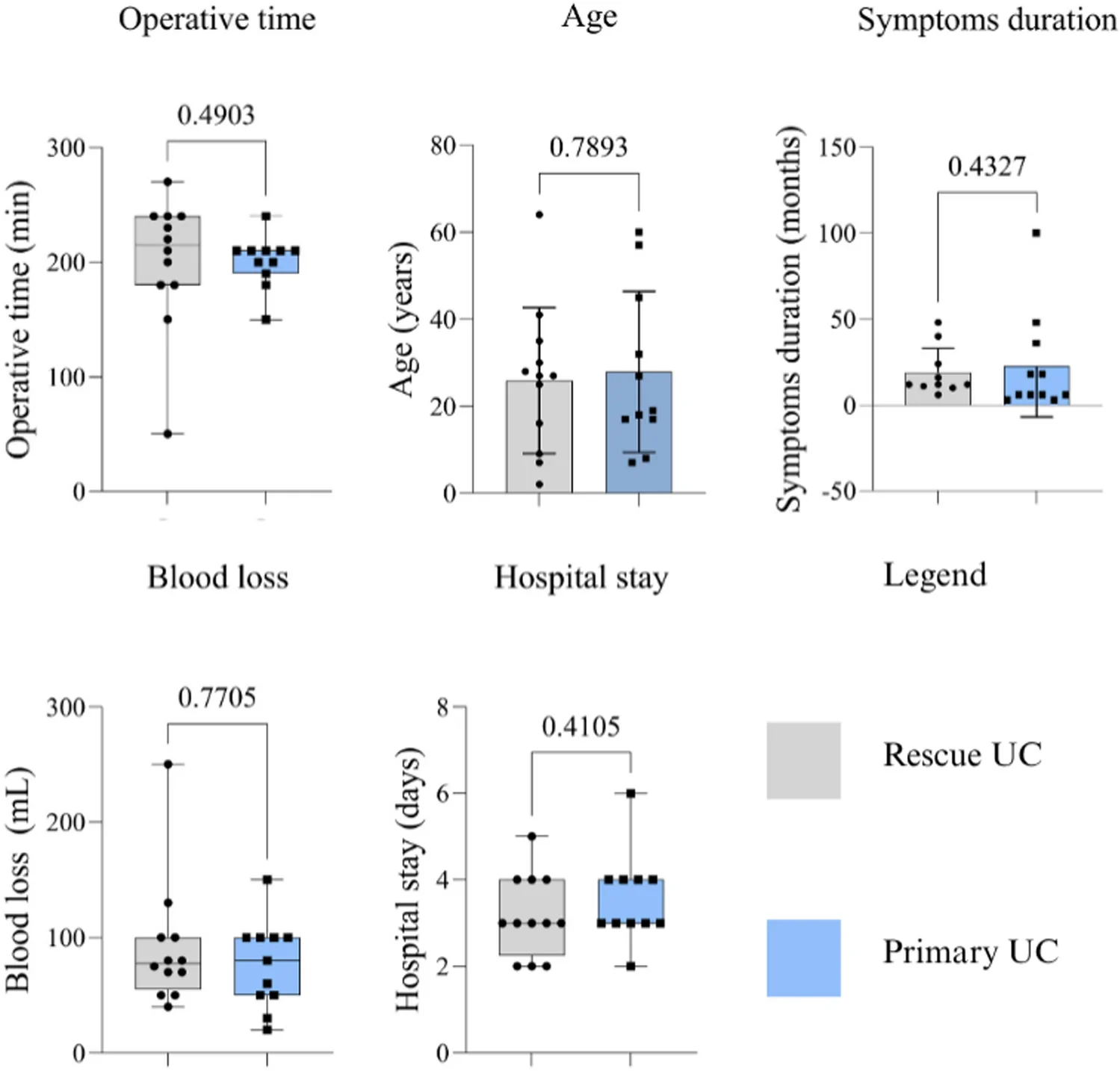
Comparison of baseline and perioperative variables between the primary and rescue groups (age, symptom duration, operative time, estimated blood loss, and hospital stay). Differences were not statistically significant. (*UC* ureterocalicostomy)

**Fig. 2:**
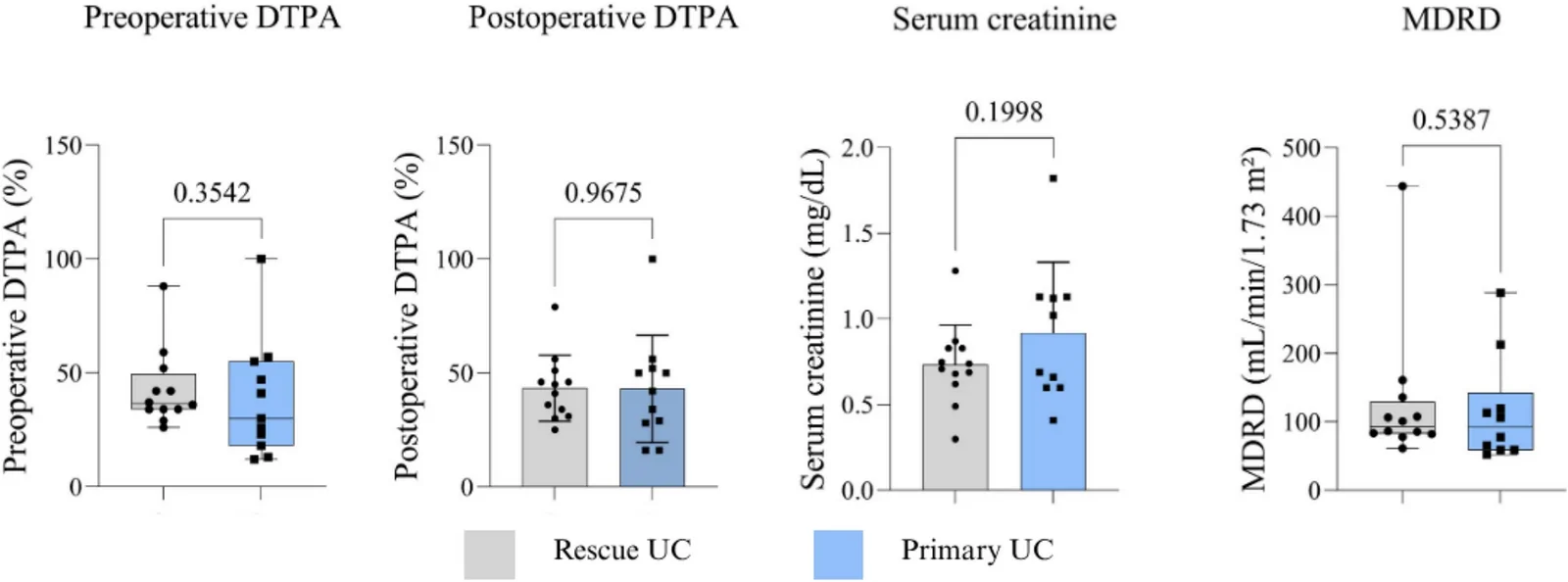
Comparison of renal function between the primary and rescue groups: preoperative and postoperative split renal function (*DTPA*), serum creatinine, and estimated glomerular filtration rate (*MDRD*)

**Fig. 3:**
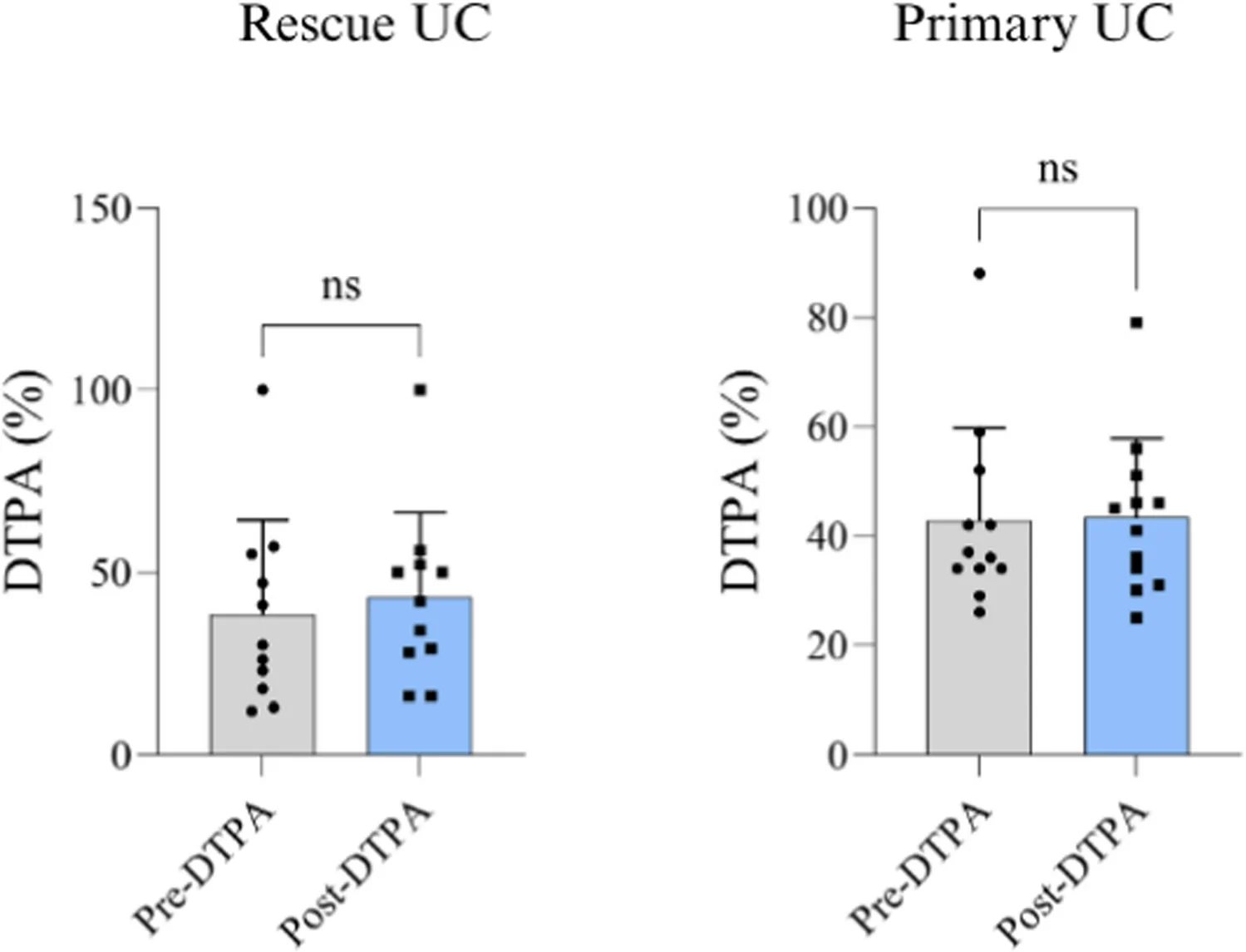
Split renal function (DTPA, % uptake by the operated kidney) before and after laparoscopic ureterocalicostomy

The primary and rescue groups were comparable across all baseline and perioperative variables: age (*p* = 0.79), symptom duration (*p* = 0.43), operative time (*p* = 0.49), estimated blood loss (*p* = 0.77), and hospital stay (*p* = 0.41) (Fig. 1). Renal function was likewise comparable between groups, both before surgery (split function *p* = 0.35) and after surgery (split function *p* = 0.97; serum creatinine *p* = 0.20; eGFR *p* = 0.54) (Figs. 1 and 2). Within each group, split renal function did not change significantly from before to after surgery in either the primary or the rescue setting (Fig. 3).

After a median follow-up of 60 months (mean 65; range 10–156), 14 patients (58%) had been followed for at least 5 years and 6 patients for at least 9 years. Twenty-two patients (91.6%) achieved both clinical and radiological resolution of the obstruction, with no symptoms, infections, or further procedures among those with the longest follow-up.

For the cohort as a whole, split renal function of the operated kidney was 40.0 ± 20.6% before surgery and 43.2 ± 18.5% afterward; 8 patients (33%) had a preoperative split function below 30% (as low as 12%), and two kidneys were effectively solitary or dominant (preoperative split function of 88% and 100%). Function of the operated kidney improved in 7 patients, remained stable in 11, and worsened in 5; the excluded patient had no postoperative scintigraphy after nephrectomy.

**Fig. 4:**
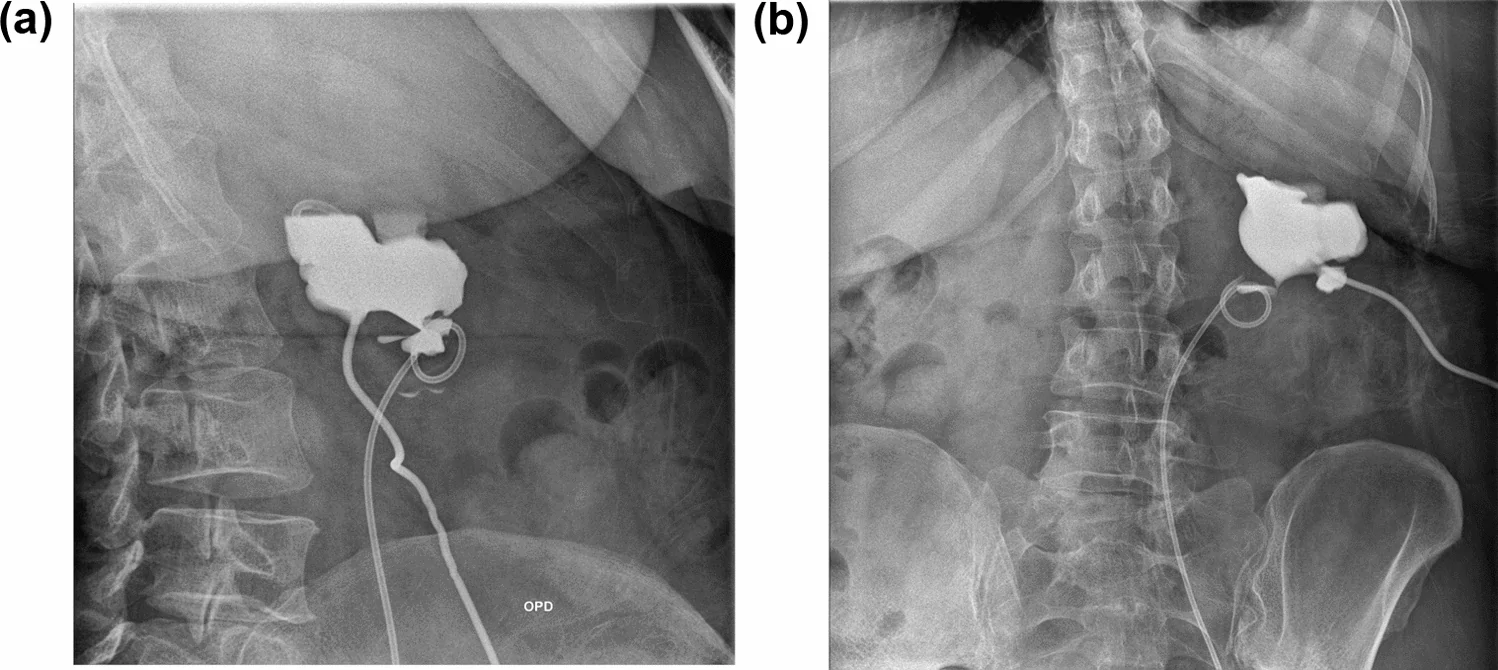
Antegrade pyelography through the nephrostomy tube, lateral (**a**) and anteroposterior (**b**) views, demonstrating infundibular stenosis of the lower calyx, with the double-J stent in the lower calyx and the nephrostomy tube in the middle calyx and no opacification of the lower calyx

**Fig. 5:**
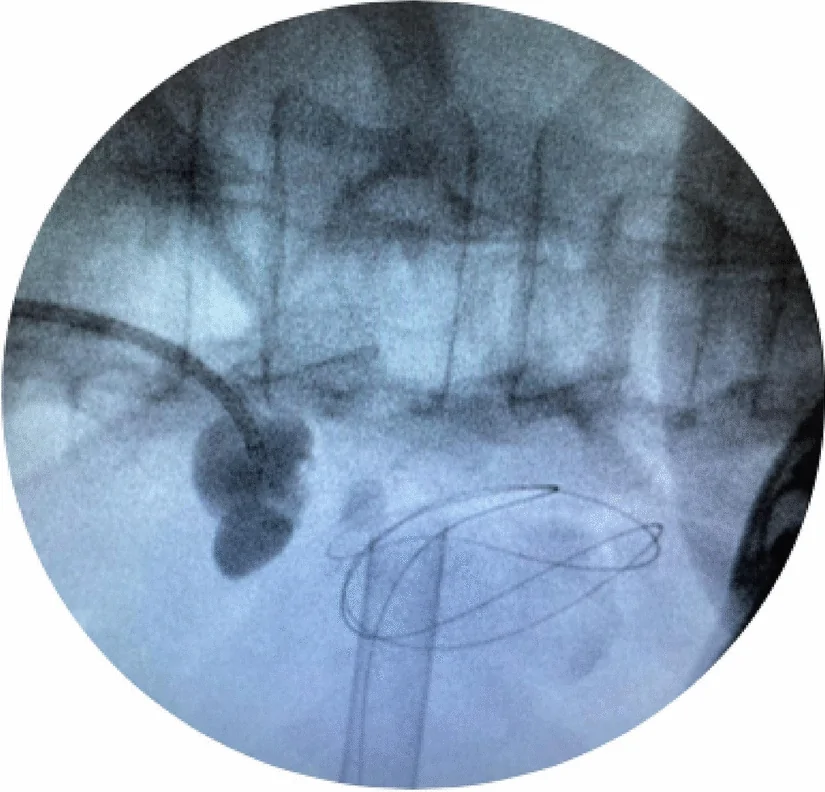
Intraoperative fluoroscopy showing the unsuccessful attempt at endoscopic and percutaneous infundibulotomy because of a blind-ending lower calyx

The two adverse outcomes were related to complex calyceal anatomy. In the primary group, one patient developed pain and restenosis 18 months after surgery, confirmed on scintigraphy, and is awaiting endoscopic evaluation. The single failure occurred in the excluded patient—the secondary ureteropelvic junction stenosis after multiple prior urological procedures—in whom symptoms did not improve and an infundibular stricture of the lower calyx was later identified as the cause. This patient had a history of multiple stone procedures, including percutaneous nephrolithotripsy, and secondary UPJ obstruction; a contrast allergy had limited preoperative CT evaluation, and ureterocalicostomy had been chosen because of a high stricture and a malrotated kidney. A complicated postoperative urinary tract infection required nephrostomy and intravenous antibiotics. Antegrade pyelography (Fig. 4) demonstrated infundibular stenosis of the lower calyx; an attempt at endoscopic infundibulotomy (endoscopic incision of the stenotic calyceal infundibulum) failed because of a blind-ending lower calyx (Fig. 5), and the patient elected nephrectomy rather than open revision.

## Discussion

In this single-institution series—the second largest reported for laparoscopic ureterocalicostomy and, to our knowledge, the one with the longest median follow-up—ureterocalicostomy provided durable long-term relief of obstruction whether it was performed as the first reconstruction of an unoperated obstruction or as a rescue after a failed repair, although our sample is too small to establish that the two settings are equivalent. Almost all patients achieved a patent anastomosis and durable symptom resolution at a median of 60 months, and split renal function remained stable in both groups. Interpreted cautiously, these findings suggest that primary ureterocalicostomy can be a reasonable option in carefully selected patients when pyeloplasty is not feasible, rather than a procedure confined to rescue.

The feasibility of the laparoscopic approach was first shown in a porcine survival model by Cherullo et al. in 2003 [15] and then clinically by Gill et al. in 2004 [2]; the robotic technique followed in 2007 [3] and 2009 [16]. For many years, however, the literature consisted almost entirely of single- or two-case reports describing feasibility [2, 15, 17]. Our 2014 series of six patients was the largest at the time, with complete symptom and radiological resolution [9]. Larger experiences followed [11–13]. In 2017, Srivastava et al. reported the largest series to date—38 laparoscopic and 34 open cases—with a 69.5% success rate at 60.3 months, one of the lowest reported [10], whereas other laparoscopic [11, 13] and robotic [18] series have reported success above 90%. Our results are consistent with the latter, with comparable success and longer follow-up.

Indications for ureterocalicostomy are uncommon, and in our practice, the decision was always made intraoperatively, when intense fibrosis, a long stricture, or giant hydronephrosis with a small intrarenal pelvis prevented an adequate tension-free pyeloplasty. Redo pyeloplasty and endopyelotomy remain alternatives in recurrent stenosis, but dissection of the upper ureter can be very difficult and our medium-term experience with endopyelotomy has been unfavorable [19]. The present data suggest that the same reasoning extends to the previously unoperated kidney: rather than persisting with a hazardous pyeloplasty in unfavorable anatomy, conversion to ureterocalicostomy offers reliable function and durable drainage. Conversely, ureterocalicostomy is ill-advised when the lower-pole parenchyma is not sufficiently thinned to allow safe amputation without vascular clamping, or when the calyceal anatomy is unfavorable or has not been fully characterized, because a stenotic or blind-ending infundibulum may doom the anastomosis—the mechanism of the single failure in this series.

Parenchymal thickness is a key consideration. Ideally, lower-pole resection is performed without vascular clamping [9]. One study identified an inferior-pole parenchymal thickness below 10 mm as the strongest predictor of effectiveness [20], while another found that a cortex thinner than 5 mm and a low preoperative glomerular filtration rate (below 20 mL/min/1.73m^2^) independently predicted failure [10]. In our series, the surgeons reviewed CT images to confirm adequate thinning before proceeding. Notably, both unfavorable outcomes were driven by complex calyceal anatomy: a symptomatic restenosis in the primary group and, most strikingly, the single failure (the excluded secondary, post-stone stenosis), in whom a contrast allergy had precluded a complete preoperative calyceal assessment and a blind-ending lower calyx was recognized only afterward. This pattern reinforces, rather than weakens, the central message: extending ureterocalicostomy to the primary setting is safe only when the calyceal anatomy is fully characterized beforehand, ideally with contrast-enhanced CT.

Our study has limitations. It is retrospective and has no control group; given the rarity of the procedure and the lack of good alternatives in these patients, a randomized comparison would be difficult to assemble. Group allocation reflects clinical history rather than randomization. The sample is small (12 vs 11 patients) and the study is not powered to demonstrate formal equivalence or non-inferiority; the comparisons should therefore be read as hypothesis-generating, and the absence of a detected difference does not prove that none exists. The cohort is also heterogeneous—spanning children and adults, primary and redo cases, varied anatomy, and two anastomotic techniques (termino-terminal and termino-lateral)—which further limits subgroup interpretation. Objective anatomical parameters were only partially available, as several children were assessed by ultrasonography alone and some older imaging could not be retrieved. With only two adverse events, the 91.6% success estimate is fragile. First, the criterion used to classify functional change (a 5-point shift in split function) is insensitive and potentially misleading in poorly functioning kidneys, in which a small absolute change may be clinically important or, conversely, within measurement noise; because one third of kidneys had a preoperative split function below 30%, the functional categories should be interpreted cautiously. Second, the choice of reconstruction over nephrectomy relied on the operating surgeon's judgment rather than a predefined threshold, introducing selection bias that may limit reproducibility outside high-volume centers. Larger, prospective, multicenter studies with predefined objective selection criteria are still needed.

Within these limits, our long-term data indicate that patients with a history of UPJ surgery and selected patients with renal anomalies that prevent safe access to the proximal ureter at a first operation are appropriate candidates for laparoscopic ureterocalicostomy. In experienced hands, the procedure offers good outcomes that persist over the long term in both settings.

## Conclusion

In this series with a median follow-up of 60 months, laparoscopic ureterocalicostomy was associated with a low complication rate (no conversions and no major intraoperative complications) and durable resolution of the obstruction in 91.6% of patients. Outcomes did not differ detectably between the primary and rescue procedures, although the study is underpowered to establish equivalence; the findings support considering primary ureterocalicostomy as a reasonable option in carefully selected patients when pyeloplasty is not feasible, rather than restricting it to the rescue setting. The two adverse outcomes related to complex calyceal anatomy underline that careful preoperative assessment of the calyces—ideally with contrast-enhanced CT—is essential to patient selection.

## Data Availability

The datasets generated and analysed during the current study are not publicly available because they contain information that could compromise the privacy of individual patients, but are available from the corresponding author (H.L.M.) on reasonable request.

## References

[1] Neuwirt K (1948) Implantation of the ureter into the lower calyx of the renal pelvis. Urol Cutaneous Rev 52(6):351 18871083

[2] Gill IS, Cherullo EE, Steinberg A, Desai MM, Abreu SC, Ng C et al (2004) Laparoscopic ureterocalicostomy: initial experience. J Urol 171(3):1227–1230 10.1097/01.ju.0000114233.66534.b0 14767308

[3] Korets R, Hyams ES, Shah OD, Stifelman MD (2007) Robot-assisted laparoscopic ureterocalicostomy. Urology 70(2):366–369 10.1016/j.urology.2007.04.024 17826511

[4] Rohrmann D, Snyder HM 3rd, Duckett JW Jr, Canning DA, Zderic SA (1997) The operative management of recurrent ureteropelvic junction obstruction. J Urol 158:1257–1259 10.1097/00005392-199709000-00154 9258188

[5] Selli C, Rizzo M, Moroni F, Dedola G, Amorosi A (1992) Ureterocalicostomy in the treatment of pyeloplasty failures. Urol Int 48:274–277 10.1159/000282350 1589916

[6] Xie B, Wang X, Zeng X, Xie L, Zeng Z, Xu H (2024) Ureterocalicostomy for complex upper ureteral stricture: a narrative review of the current literature. Int Urol Nephrol 56(6):1899–1909 10.1007/s11255-023-03911-8 PMC11090920 38252259

[7] Osman T, Eltahawy I, Fawaz K, Shoeib M, Elshawaf H, El Halaby R (2011) Ureterocalicostomy for treatment of complex cases of ureteropelvic junction obstruction in adults. Urology 78:202–207 10.1016/j.urology.2011.01.044 21439615

[8] Mesrobian HG, Kelalis PP (1989) Ureterocalicostomy: indications and results in 21 patients. J Urol 142:1285–1287 10.1016/s0022-5347(17)39058-4 2810508

[9] Arap MA, Andrade H, Torricelli FC, Denes FT, Mitre AI, Duarte RJ, Srougi M (2014) Laparoscopic ureterocalicostomy for complicated upper urinary tract obstruction: mid-term follow-up. Int Urol Nephrol 46(5):865–869 10.1007/s11255-013-0591-z 24217801

[10] Srivastava D, Sureka SK, Yadav P, Bansal A, Gupta S, Kapoor R et al (2017) Ureterocalicostomy for reconstruction of complicated ureteropelvic junction obstruction in adults: long-term outcome and factors predicting failure in a contemporary cohort. J Urol 198(6):1374–1378 10.1016/j.juro.2017.06.079 28652124

[11] Ansari MS, Danish N, Yadav P, Kaushik VN, Kakoti S, Kumar A et al (2021) Role of ureterocalicostomy in management of giant hydronephrosis in children in contemporary practice: indications, outcomes and challenges. J Pediatr Urol 17(5):657.e1-e7 10.1016/j.jpurol.2021.06.007 34176750

[12] Mittal S, Aghababian A, Eftekharzadeh S, Saxena S, Janssen K, Lombardo A et al (2022) Robot-assisted laparoscopic ureterocalicostomy in the setting of ureteropelvic junction obstruction: a multi-institutional cohort. J Urol 208(1):180–185 10.1097/JU.0000000000002484 35188821

[13] Esposito C, Blanc T, Patkowski D, Lopez PJ, Masieri L, Spinoit AF et al (2022) Laparoscopic and robot-assisted ureterocalicostomy for treatment of primary and recurrent pelvi-ureteric junction obstruction in children: a multicenter comparative study with laparoscopic and robot-assisted Anderson-Hynes pyeloplasty. Int Urol Nephrol 54(10):2503–2509 10.1007/s11255-022-03305-2 PMC9463286 35861906

[14] Nunes RSS, Suartz CV, Andrade HS, Jordao RD, Srougi V, Mitre AI et al (2023) Laparoscopic ureterocalicostomy technique. Int Braz J Urol 49(4):517–518 10.1590/S1677-5538.IBJU.2022.0521 PMC10482455 37267617

[15] Cherullo EE, Gill IS, Ponsky LE, Banks KL, Desai MM, Kaouk JH et al (2003) Laparoscopic ureterocalicostomy: a feasibility study. J Urol 169:2360–2364 10.1097/01.ju.0000058214.99086.37 12771798

[16] Schimpf MO, Wagner JR (2009) Case report: robotic-assisted laparoscopic ureterocalicostomy with long-term follow-up. J Endourol 23:293–295 10.1089/end.2008.0165 19220087

[17] Kumar S, Panigrahy B (2009) Laparoscopic management of complex ureteropelvic junction obstruction. J Laparoendosc Adv Surg Tech A 19:521–528 10.1089/lap.2008.0397 19645600

[18] Ramanitharan M, Lalgudi Narayanan D, Sreenivasan SR, Sidhartha K, Mehra K, Rajiv K et al (2020) Outcomes of robot-assisted ureterocalicostomy in secondary ureteropelvic junction obstruction in adults: initial experience using the da Vinci Xi system with near-infrared fluorescence imaging. J Laparoendosc Adv Surg Tech A 30(1):48–52 10.1089/lap.2019.0531 31794685

[19] Brito AH, Mitre AI, Arap S (2002) Endopyelotomy with the Acucise catheter. Int Braz J Urol 28:302–309 (**discussion 309–310**) 15748335

[20] Demchenko V, Shchukin D, Antonyan I, Lisova G, Harahatyi A, Shus A (2020) Ureterocalicostomy for reconstruction of the upper urinary tract. Georgian Med News 303:33–39 32841177

